# Phospho-RNA sequencing with circAID-p-seq

**DOI:** 10.1093/nar/gkab1158

**Published:** 2021-12-01

**Authors:** Alessia Del Piano, Tea Kecman, Michael Schmid, Ruggero Barbieri, Luciano Brocchieri, Silvia Tornaletti, Claudia Firrito, Luca Minati, Paola Bernabo, Ilaria Signoria, Fabio Lauria, Thomas H Gillingwater, Gabriella Viero, Massimiliano Clamer

**Affiliations:** IMMAGINA BioTechnology S.r.l, Via Sommarive 18, Povo, Italy; Department of Cellular, Computational and Integrative Biology (CIBIO), University of Trento, Trento, Italy; IMMAGINA BioTechnology S.r.l, Via Sommarive 18, Povo, Italy; Genexa AG, Zurich, Switzerland; IMMAGINA BioTechnology S.r.l, Via Sommarive 18, Povo, Italy; TB-Seq, Inc., 458 Carlton Court, Ste H, South San Francisco, CA 94080, USA; TB-Seq, Inc., 458 Carlton Court, Ste H, South San Francisco, CA 94080, USA; IMMAGINA BioTechnology S.r.l, Via Sommarive 18, Povo, Italy; IMMAGINA BioTechnology S.r.l, Via Sommarive 18, Povo, Italy; IMMAGINA BioTechnology S.r.l, Via Sommarive 18, Povo, Italy; Institute of Biophysics, Unit at Trento, CNR, Via Sommarive, 18 Povo, Italy; Institute of Biophysics, Unit at Trento, CNR, Via Sommarive, 18 Povo, Italy; Edinburgh Medical School: Biomedical Sciences & Euan MacDonald Centre for Motor Neurone Disease Research, University of Edinburgh, Edinburgh, UK; Institute of Biophysics, Unit at Trento, CNR, Via Sommarive, 18 Povo, Italy; IMMAGINA BioTechnology S.r.l, Via Sommarive 18, Povo, Italy

## Abstract

Most RNA footprinting approaches that require ribonuclease cleavage generate RNA fragments bearing a phosphate or cyclic phosphate group at their 3′ end. Unfortunately, current library preparation protocols rely only on a 3′ hydroxyl group for adaptor ligation or poly-A tailing. Here, we developed circAID-p-seq, a PCR-free library preparation for selective 3′ phospho-RNA sequencing. As a proof of concept, we applied circAID-p-seq to ribosome profiling, which is based on sequencing of RNA fragments protected by ribosomes after endonuclease digestion. CircAID-p-seq, combined with the dedicated computational pipeline circAidMe, facilitates accurate, fast and highly efficient sequencing of phospho-RNA fragments from eukaryotic cells and tissues. We used circAID-p-seq to portray ribosome occupancy in transcripts, providing a versatile and PCR-free strategy to possibly unravel any endogenous 3′-phospho RNA molecules.

## INTRODUCTION

RNA molecules bearing a phosphate or cyclic phosphate group at the 3' end (3′P/2′-3′cP) are generated by either heat fragmentation (1), ribonucleases (e.g. RNase A superfamily) (2), ribozymes (3,4) or toxins (5,6). Beside endogenous 3′P/2′-3′cP RNA molecules (7–11), several biochemical methodologies designed to obtain genome wide positional information of RNA-protein interaction require an enzymatic digestion step to generate phosphorylated 3' termini prior to library preparation and RNA sequencing. This is the case of most RNA footprinting protocols used to understand the global regulatory network underlying protein and RNA fate in living cells (12). These protocols are based on (i) RNA:protein cross-linking and immunoprecipitation (CLIP-seq) (13–15) (ii) selective RNA:protein immunoprecipitation (RIP-seq) (16,17), (iii) RNA:protein affinity purification (uvCLAP) (18) and (iv) ribosome profiling (Ribo-seq). Commonly used enzymes for RNA footprinting include those belonging to the RNase A superfamily (e.g. RNase I) (19), RNase T1 (20) and RNase T2 (21), which produce 3′ phosphate or cyclic phosphate RNA molecules. No technologies are currently available to directly sequence 3′P/2′-3′cP RNA fragments, without removing the 3′P/2′-3′cP by enzymatic reactions before library preparation. Methods that provide insights into endogenously generated 3′-phospho RNA species rely on an indirect detection of these fragments by means of a periodate treatment (22) and downstream stringent bioinformatics analysis (23). However, these approaches introduce potential sequencing biases related to 3′ de-phosphorylation (24) and PCR amplification steps (25,26), and are time-consuming and computationally expensive.

Here, we present circAID-p-seq (CIRCular Amplification and IDentification of short 3′ Phosphate RNA SEQuences) a library preparation method uniquely characterized by the selection of 3′P/2′-3′cP terminated RNA fragments and cDNA synthesis by rolling circular amplification (RT-RCA) (27,28). The RT-RCA has the advantage of not requiring any additional PCR amplification, and it produces a long (>200 nt), single-stranded cDNA molecule bearing multiple copies of a unique RNA fragment. This cDNA is suitable for direct cDNA sequencing with Oxford Nanopore Technologies (ONT) instruments.

Ribo-seq can be considered an effective case study to test circAID-p-seq, because it requires the sequencing of 25–35 nt long ribosome-protected fragments (RPFs) produced by 3′-P/cP generating nucleases (e.g. RNase I or the Micrococcal). To evaluate the performance of our new library construction method, we compared our results to well-known library preparation and sequencing approaches (29,30). To our knowledge, this is an original method for 3′-P/cP RNA-seq library preparation and the first selective 3′-P/cP ribosome profiling obtained with the Oxford Nanopore Technologies (ONT) platform.

## MATERIALS AND METHODS

### circAID-p-seq synthetic library preparation

#### 5′ phosphorylation and adaptor ligation

Synthetic RNA fragments bearing a 3′P were subjected to 5′ phosphorylation with T4 PNK 3′ minus (NEB, cat no. M0236S), according to manufacturer's instructions. RNA fragments were purified using a RNA Clean & Concentrator™-5 column (Zymo Research, cat. no. R1013). The resulting RNA fragments were ligated to various adaptors (listed in Additional file 1) via 3′P ligase (RtcB, NEB), according to the following conditions: 30 pmol of RNA fragment, 10 pmol of adaptor, 15 pmol 3′P ligase, 1 × 3′P ligase buffer, 100 μM GTP, 1 mM MnCl_2_ in a final volume of 10 μl. The reaction was incubated 2 h at 37°C and then loaded on a 15% acrylamide/8M urea precast gel (Invitrogen, cat no. EC6885BOX). The ligated RNA was purified through gel extraction, as described in the ‘RNA extraction from TBE urea gel’ section below. To evaluate and optimize the circAID-p-seq method, different combinations of adaptors and synthetic RNA fragments were employed. For testing the effect of adaptor lengths on RT-RCA and number of repetition, different adaptors (named ADR-110, ADR-60, ADR-20) were used for ligation with a 30 nt long synthetic RNA molecule bearing a 3′P group (30RNA-3′P). Subsequently, to identify the best sequence for the 24 nt long adaptor, an equimolar pool of 12 oligos was produced and used in the first ligation step with 30RNA3′P fragment. For quantitative analysis, three different RNA fragments (named RNA30-G, RNA30-M, RNA30-A) were combined at the ratio of 1:10:100. In this case, for the circAID-p-seq library preparation, the adaptor ADR-12 was used.

#### Circularization and RNase R treatment

The circularization of the adaptor-ligated RNA (RNA:adaptor) was carried out at 25°C for 2 h, in a total volume of 20 μl containing 10 U of T4 RNA Ligase 1 (NEB, cat. no. M0204L), 1× T4 RNA ligase buffer, 20% PEG8000, 50 μM ATP. The reaction was then incubated at 37°C for 1 h with 20 U of RNase R (Lucigen, cat. no. RNR07250), to remove undesired products (i.e. linear RNA or concatemer product). Circular RNA was purified by using RNA Clean & Concentrator™-5 column (Zymo research, cat. no. R1013) and quantified using Qubit™ RNA HS Assay Kit (Thermo Fisher, cat. no. Q32852).

#### Reverse transcription-rolling circle amplification (RT-RCA) and second strand synthesis

RT-RCA reaction was performed in 20 μl final volume, with Maxima H Minus Reverse Transcriptase (Thermo Fisher, cat. no. EP0752) under the following conditions: 50 ng of circular RNA, 200 U of Reverse Transcriptase, 1× RT buffer, 0.5 mM dNTPs, 50 pmol Primer ADRX_R (additional file 1), 10% glycerol. The reaction was carried out at 42°C for 4 h, then stopped by incubation at 70°C for 10 min. After cDNA synthesis, circular RNA template was hydrolyzed by adding 0.1 N NaOH for 10 minutes at 70°C. The second strand cDNA was produced by performing one PCR cycle using Platinum II Hot start Taq Polymerase (Thermo Fisher, cat. no. 14966001). The reaction included 20 μl of single-strand cDNA, 1× buffer, 0.2 mM dNTPs, 2 mM MgCl_2_, 2 U Taq Polymerase, 50 pmol primer ADRX_F (Additional file 1) in a total volume of 50 μl. The mix was then subjected to the following conditions for second strand cDNA synthesis: initial denaturation at 94°C, one cycle of 94°C for 30 s, 60°C for 30 s and 68°C for 2 min. Double strand cDNA was purified using AMPure XP beads (Agencourt, cat. no. A63881) according to manufacturer's instructions. Validation of second strand synthesis was performed by Nuclease S1 digestion (Thermo Fisher, cat. no. EN0321) according to manufacturer's instructions.

#### Nanopore sequencing

Purified double-strand cDNA was prepared for nanopore sequencing according with ONT protocol for cDNA sequencing (SQK-DCS109). Briefly, cDNA was subjected to end repair and dA-tailing reaction using NEBNext End repair/dA-tailing module (NEB, cat. no. E7546S) following the manufacturer's instruction and incubated for 5 min at 20°C and then 5 min at 65°C. The reaction mix was purified with AMPure XP beads (Agencourt). ONT Adaptor mix was added according to the direct cDNA sequencing kit protocol (SQK-DCS109, ONT), then loaded on a R9.4 flow cell and sequenced in the MinION sequencing device. The cDNA sequencing kit recommends an input of 100 ng poly-A+ RNA that should generate an output of about 5–10 million reads per flow cell. In all our experiments we started with 50–120 ng of RPF extracted from TBE-urea gel. Not all fragments purified from the gel are 3′phosphorylated (data not shown) and therefore cannot be selected by circAID in the first ligation, suggesting that only 25–40 ng of phosphorylated RNA can be used for the library. After final library check by Qubit™ small RNA Assay Kits (Thermo Fisher) the recovery is around ∼10–25 ng, about half of expected. This explain the lower (2–5 million reads/sample) than expected (5–10 million reads/sample) in our experiments compared to manufacturer's guidelines.

### RNA extraction from TBE urea gel

RNA samples were mixed 1:1 with gel loading II (Thermo Fischer Scientific, cat no. AM8547), denatured at 70°C for 90 s before being loaded into the TBE–urea gel and run at 200 V. Gels were then stained with Sybr™ Gold (Invitrogen, cat. no. S11494) and scanned using Chemidoc (GE Healthcare, Piscataway, NJ). Gel images were analyzed using ImageLab (Biorad). When required, bands were isolated from the gel, crushed and soaked overnight in Buffer I (Immagina Biotechnology, cat. no. RL001-10) at room temperature with constant rotation. The aqueous gel debris was filtered with Millipore ultrafree MC tubes and then precipitated with isopropanol (Sigma, cat. no. I9516) at −80°C for 2 h or overnight. After precipitation, samples were centrifuged for 30 min at 12 000 g, 4°C. The pellet was washed once with 70% ethanol, centrifuged at 12 000 g for 5 min at 4°C and air-dried before further processing.

### Synthetic oligonucleotides

Custom RNA adaptors, synthetic RNA fragments and DNA primers were purchased from Integrated DNA Technologies (Coralville, IA). Synthetic RNA fragments consisted of 30-mer oligonucleotides with 5′OH and 3′P termini. Custom adaptors consisted of RNA molecules with different lengths and no modification at 5′ and 3′ ends. Adaptors were carefully designed to minimize their secondary structure, using a combination of RNAFold and OligoEvaluator folding tools. All sequences are listed in Additional file 1.

### Cell culture and treatments

HEK293 (*H. sapiens*, RRID: CVCL_0045) cells were transfected with a plasmid encoding GFP (pMAX_GFP, Lonza). GFP expression was monitored by fluorescence microscopy (Olimpus IX-50). After 24 h after transfection, cells were treated with harringtonine (2 μg/ml) for 3 min, followed by cycloheximide addition (CHX, 10 μg/ml, SIGMA cat. no. 01810) and incubation for 5 min at 37°C. Controls were not treated with harringtonine. Cell lysates were obtained using a hypotonic lysis buffer (IMMAGINA Biotechnology, cat. no. RL001-1). Lysate absorbance at 260 nm was measured by Nanodrop ND-1000 UV-VIS Spectrophotometer and then diluted to a final value of 1.7 a.u. (absorbance measured at 260 nm) in 250 μl of W-buffer (IMMAGINA Biotechnology, cat. no. RL001-4). Ribosome protected Fragments (RPFs) were generated by treating the diluted lysate with 12.7 U of RNase I (Ambion, cat. no. AM2295) at room temperature for 45 min in an orbital mixer (31). RNase I digestion was stopped by adding 10U of Supernase Inhibitor (Thermo Scientific, cat. no. AM2696) for 10 min on ice.

### Sucrose cushioning of HEK293T cell lysates

After RNAse I digestion, HEK293T cell lysates were loaded on top of 900 μl of a 30% sucrose cushion (30 mM Tris–HCl pH 7.5, 100 mM NaCl, 10 mM MgCl_2_, 1 M sucrose in nuclease-free water) supplemented with 20 μg/ml of CHX. Samples were ultracentrifuged at 95 000 rpm at 4°C for 2 h using a TLA100.2 rotor (Beckman). The pellets were resuspended in 200 μl of W-Buffer and treated with 1% SDS (Sigma cat. no. 05030) and 0.1 mg Proteinase K (Euroclone, cat. no. EMR022001) at 37°C for 75 min. RNA was extracted by acid-phenol:chloroform, pH 4.5 (Ambion, cat. no. AM9722), precipitated with isopropanol, air-dried, resuspended in nuclease-free water and analyzed on 15% acrylamide/8M urea precast gel. RPFs were size-selected (corresponding to 25–35 nt bands) and extracted from gel. Before starting with library preparation, isolated and purified RPFs were quantified using the Qubit™ miRNA Assay Kit (Thermo Fisher, cat. no. Q32881).

### Purification of ribosome protected fragments from mouse liver

Mouse liver were dissected immediately following sacrifice of wild-type FVB mice obtained from breeding stocks at the University of Edinburgh. All procedures were performed under licensed authority from the UK Home Office (PPL P92BB9F93). Tissues were pulverized under liquid nitrogen using a pestle and a mortar and the lysates were obtained according to previous protocols (32). Lysates were treated with RNAse I and the 80S were isolated using sucrose gradient separation according to previous protocols (33). The RNA were purified using acidic phenol/chloroform extraction from the 80S sucrose fraction. RPFs (25–35 nt) were obtained after purification in denaturing 15% UREA-PAGE and divided into two aliquots. One was used for library preparation according to Ingolia protocol (30,33). The other aliquot was used for circAID-p-seq. All experiments were performed in biological triplicate.

### Library preparation for Ribosome profiling experiment

#### Illumina library preparation

Libraries from RPFs isolated from HEK293T cells were prepared using the SMARTer^®^ smRNA-Seq Kit for Illumina (Takara, cat. no. 635029) and sequenced with 50 cycles single-read on an Illumina NovaSeq 6000. HEK293T RPFs were extracted from independent biological replicates for circAID-p-seq/ONT and SMARTer^®^ smRNA-Seq /Illumina sequencing. Lists of the counts per gene are reported in Additional file 2. Mouse liver RPFs were extracted from three biological replicates. For each replicate, the same pool of PAGE purified RPFs of each triplicate were used for parallel circAID-p-seq/ONT and Illumina sequencing. Illumina libraries for mouse liver were prepared according to Ingolia et al., 2012 (30) and sequenced with 50 cycles single-read on an Illumina HiSeq2500. List of the counts per gene are reported in Additional file 3.

#### CircAID-p-seq library preparation

CircAID-p-seq library preparation for both HEK293T and mouse liver samples, was performed following the protocol for circAID-p-seq as described above.

In particular, upon RPFs isolation, 5′ phosphorylation was performed with T4 PNK 3′ minus (NEB, cat. no. M0236S), according to manufacturer's instructions. RNA fragments were purified from the reaction using a RNA Clean & Concentrator™-5 column (Zymo Research, cat. no. R1013). The resulting RNA fragments were ligated via 3′P ligase (RtcB, NEB), according to the following conditions: 30 pmol of RNA fragment, 10 pmol of ADR12, 15 pmol 3′P ligase, 1 × 3′P ligase buffer, 100 μM GTP, 1 mM MnCl_2_ in a final volume of 10 μl. The reaction was incubated 2 h at 37°C and then purified through gel extraction, as described in the gel analysis section above. After gel purification, ligation product (RPFs:ADR12) was subjected to circularization, followed by RNase R treatment according to the reaction condition described in the paragraph above (Circularization and RNAse R treatment). Circular RNA was purified through RNA Clean & Concentrator™-5 column (Zymo research, cat. no. R1013), then subjected to RT-RCA and second strand synthesis according to the reaction condition described in the paragraph above (reverse transcription-rolling circle amplification (RT-RCA) and second strand synthesis). In particular, 50 pmol of ADR12_R and 50 pmol of ADR12_F primers (Additional file1) were used for RT-RCA reaction and second strand synthesis, respectively. Double strand cDNA was purified using AMPure XP beads (Agencourt, cat. no. A63881) according to manufacturer's instructions and then subjected to end repair and dA-tailing reaction using NEBNext End repair/dA-tailing module (NEB, cat. no. E7546S) following the manufacturer's instruction. The reaction mix was purified with AMPure XP beads (Agencourt). ONT Adaptor mix was added according to the direct cDNA sequencing kit protocol (SQK-DCS109, ONT), then loaded on a R9.4 flow cell and sequenced for 20–24 h with MinION device.

### circAID-p-seq data analysis with CircAidMe

CirAID-p-seq produces long concatemeric molecules containing tandem repeats; each repeat comprises one adaptor and one fragment of interest (called insert hereinafter). Once the cDNA is produced, it can be sequenced with the ONT platform. In ribosome profiling experiments, each insert is a ribosome protected fragment. To analyse the data obtained with cirAID-p-seq we developed a custom pipeline (written in Python 3): CircAidMe. The FASTQ files obtained by the Guppy 3.6.1 (available from ONT via https://community.nanoporetech.com) base calling are processed to generate the consensus sequence of the RNA inserts within each selected concatemeric read. The selection criteria are defined by the user and include filtering by insert length (for our libraries we selected fragment lengths between 15 and 40 nt), read length and by minimum number of RNA inserts in the concatemeric reads.

For quantitative analysis, we first reasoned that the two strands of the concatemeric cDNA carry complementary information (Supplementary Figure S4A). If this was the case, only one strand of the double stranded cDNA should to be taken into account for an accurate quantitative analysis and for avoiding double counting the same fragment. To evaluate if this had occurred, and to determine which strand is the most reliable, we compared the raw read length of both forward and reverse cDNA concatemeric strands based on the orientation of the adaptor sequence (Supplementary Figure S4A). We noticed that forward reads, generated during the second strand synthesis, are generally shorter and less abundant than the reverse reads (Supplementary Figure S4B). This effect most probably derives from multiple annealing sites of the primer for second strand synthesis on the concatemeric cDNA. Therefore, since forward-strand synthesis can generate fragmented copy of the reverse strand, the forward strand could introduce biases in quantitative experiments. Then, we noticed that a portion of the reads carry forward and reverse strands fused together. This effect is known to be caused by two different molecular reactions: (i) a second strand entering the pore immediately after the first strand without the sequencing device been able to detect two independent molecules (we called this type of reads ‘fused reads’) (34) or (ii) a hairpin at the 3′-end of the cDNA strand that functions as a primer during second strand synthesis (35,36) (we called this type of reads ‘hairpin reads’, Supplementary Figure S4A). We optimized CircAidMe to split fused reads, while leaving hairpin reads untouched. The latter are useful to produce longer reads (Supplementary Figure S4B), and a better consensus for quantitative analysis.

The circAidMe pipeline performs the following steps: first, fused reads are detected by (i) searching for remaining ONT adaptors in a read after adaptor removal and (ii) detecting orientation of the circAID-p-seq adaptors, which indicate a fused read. Fused reads are then split at the appropriate position (forward or reverse). Second, for every read the circAID-p-seq adaptors, flanking the inserts, are detected. Thus, the inserts are then identified and extracted. Third, a multiple sequence alignment (MSA) of all inserts extracted from the same read is performed with MUSCLE v3.8.1551 (37). The MSA is examined using esl-alipid (https://github.com/EddyRivasLab/easel/tree/master/miniapps) and low quality reads are detected and removed. Finally, a second MSA of the remaining inserts is performed and the consensus sequence generated. Throughout the pipeline, the detection of ONT- and circAID-p-seq adaptors is performed using SeqAn v2.4 (38).

The final consensus sequences are collected in a FASTA file for downstream analyses. Several parameters for each read are collected and stored in a CSV file while executing CircAidMe to evaluate the quality of the circAID-p-seq library, including: number of inserts per read (≥3 for Ribo-seq), number of adaptors detected per read (≥4 for Ribo-seq), read length and consensus length (≥20 nt for Ribo-seq). Moreover, reads discarded by CircAidMe are collected in a separate FASTA file and the reason of their exclusion is provided as a tag in the report file. More details about the function of CircAidMe are available at the following GitHub repository: https://github.com/ms-gx/CircAidMe.

Accuracy of the consensus sequence was determined as reported in Volden *et al.* (39). Briefly, the accuracy is represented by the portion of the consensus sequence not altered by mismatches or indels when considering the alignment to the reference sequence/coding transcriptome. The accuracy is computed for each aligned consensus sequence and expressed as a percentage:

$$\begin{eqnarray*}&&Read\ Accuracy \nonumber \\ && = \frac{{{\rm{Read\ Length}} - {\rm{ }}\left( {{\rm{Number\ of\ Mismatches}} + {\rm{Number\ of\ Insertions\ or\ Deletions}}} \right)}}{{{\rm{Read\ Length}}}}{\rm{ *}}100\end{eqnarray*}$$

### Ribosome profiling data analysis

To assess representation of different components (rRNA, tRNA, ncRNA, cDNA) of the human and mouse transcriptome in the libraries, the consensus sequences generated by CircAidMe were iteratively mapped with Bowtie2 (40) to different classes of human or mouse transcribed sequences (as annotated In Gencode v33 for human and Genecode M25 for mouse), including: rRNAs, tRNAs and other non-coding. The remaining unmapped sequences were aligned to the human (assembly GRCh38.p13) or mouse (assembly GRCm38.p6) genomes. Alignment files are processed with Samtools version 1.9 (41) and analyzed with the RiboWaltz R package (42), to identify the P-site localization within ribosome footprints, assess the three-periodicity and compute the coverage of annotated coding regions. The Read Accuracy index is calculated as previously reported (39) with a custom R script.

Illumina data from human HEK293T cells were trimmed with Cutadapt version 2.10 (43) by removing the first 3 nucleotides of each read and the 3′ terminal adaptor plus poly-A tail. Trimmed reads of length under 15 nucleotides were discarded. Illumina data from mouse liver tissue were processed according to Ingolia *et al.* (30), removing the Illumina adaptors and retaining only reads where the adaptors were present (Cutadapt parameters used: -O 15 -e 0.15 -n 3 –m 19). Illumina reads from HEK293T and mouse liver samples were analysed following the same procedure detailed above for circAID-p-seq data, using the following annotations: Gencode v33 (genome assembly GRCh38.p13) for human; Genecode vM25 (genome assembly GRCm38.p6) for mouse.

Read coverages for each protein-coding gene from ONT and Illumina libraries were calculated with HTSeq (44) and normalized as TPM values for comparison between libraries. Reads with counts > 1 were retained when comparing ONT and Illumina libraries. PCR duplicate reads from HEK293T samples were identified with Picard MarkDuplicates version 2.23.1 (Picard Toolkit. 2019. Broad Institute, GitHub Repository http://broadinstitute.github.io/picard/; Broad Institute) with default parameters, and removed with Samtools. For mouse sample all reads aligning to the very same region were collapsed. The sequencing data have been deposited in NCBI Geo database (accession GSE174754).

Gene ontology analysis was performed using EnrichR. The top-5 enriched terms for each category are selected according to the log_10_(*P*-value). The p-value is computed using Fisher's exact test. Since the Fisher's exact test produces lower *P*-values for longer lists even when the input lists are random, EnrichR precomputes a background expected rank for each term in each gene set library (45).

## RESULTS

**Figure 1.**
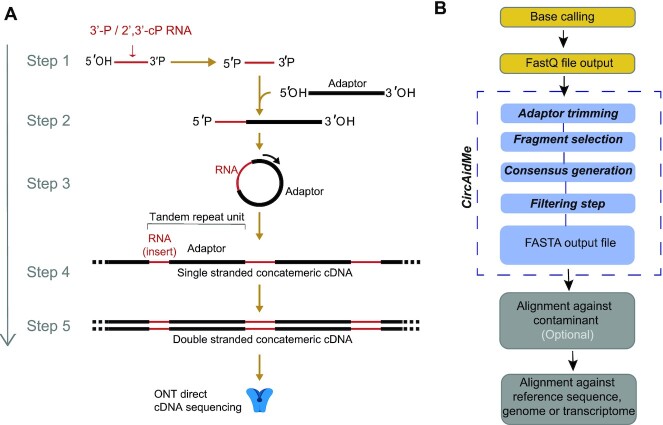
CircAID-p-seq workflow. (**A**) CircAID-p-seq library preparation: step 1: 5′ phosphorylation of the RNA fragment; step 2: selective capture of the 3′P end through the ligation with the RNA-based adaptor; step 3: circularization; step 4: Reverse Transcription - Rolling Circle Amplification (RT-RCA) - generation of the first strand, and second strand cDNA synthesis; step 5: direct cDNA nanopore sequencing. (**B**) Bioinformatics pipeline. Bases are called from the ONT output (FAST5 files) and the resulting reads (FASTQ file) are processed with circAidMe, which identifies copies of the RNA fragments and calculates their consensus sequence. Data are filtered of contaminants (e.g. rRNAs, tRNAs, other non-protein-coding transcripts) as needed and aligned against the reference sequences (e.g. genome or transcriptome).

Currently available RNA sequencing methods require 3′ RNA dephosphorylation before library preparation. To sequence 3′ phosphorylated RNAs fragments, we developed a new approach for library preparation, which proceeds according to the following five steps (Figure 1A): (i) phosphorylation of the RNA 5′ end, (ii) ligation of an RNA adaptor molecule to the 3′P or 2′,3′cP RNA ends (46), (iii) intramolecular circularization, (iv) reverse-transcription rolling circular amplification (RT-RCA) (47), (v) second strand cDNA synthesis. Importantly, the rolling circular amplification (RT-RCA) produces a long concatemeric molecule containing tandem repeats; each repeat comprises one adaptor and one fragment of interest (called insert hereinafter). Once the cDNA is produced, it can be sequenced with the ONT platform, which allows direct sequencing of the long (>100 nt) cDNA without additional PCR amplification (48). After sequencing, all copies of the RNA fragment of interest in the concatemeric raw reads can be identified and processed. For this purpose, we developed *circAidMe*, a computational pipeline implementing (i) the identification of all insert copies in the concatemeric read, (ii) multiple sequence alignment of the inserts and (iii) generation of a highly accurate consensus sequence of the RNA insert for further analyses (Figure 1B). The circAidMe python code is freely available on GitHub at: https://github.com/ms-gx/CircAidMe. To obtain meaningful biological information, consensus sequences can be mapped to the target genome or transcriptome for downstream analyses (Figure 1B). Other works used a similar linear consensus approach by means of a Phi29 polymerase rolling circle amplification on a circularized DNA, but none of them provided scientists with opportunities for sequencing reverse transcribed circular RNA to detect short RNA fragments (39,49)

To optimize our strategy, we utilized a 30 nt long synthetic RNA molecule, called RNA30-3′P, bearing a 3′P group. The phosphorylation of the 5′ terminus was carried out by the T4 polynucleotide kinase (PNK 3′-minus), a step required to block self-ligation and obtain 5′P-RNA30-3′P RNA species (Figure 1A). The ligation of the 3′P terminus to an adaptor (called ADR hereinafter, with 3′- and 5′- hydroxyl groups) was performed using a 3′P ligase (RtcB, Supplementary Figure S1A). This step produces an RNA:adaptor molecule. Intramolecular circularization of the RNA:adaptor was performed with a T4 RNA ligase to form circular RNAs. To confirm the effectiveness of the reaction and to remove remaining single stranded RNAs, the reaction mix was treated with RNase R to digest linear RNA molecules (50) (Supplementary Figure S1A). To synthesise the first cDNA strand and generate long single-stranded cDNA molecules carrying multiple copies of the insert, we performed a RT-RCA. To detect the multimeric cDNAs we synthesised the cDNA using three different reverse transcriptases and the product was amplified by PCR (Supplementary Figure S1B). Densitometric analysis of the PCR products showed an average processivity of about 500 nts on the circular RNA for all RT enzymes (Supplementary Figure S1C). Next, to synthesise the second cDNA strand we used a Taq polymerase with 5′-3′ exonuclease activity and a primer complementary to the adaptor used in the first ligation,. The efficiency of the second strand synthesis was confirmed by the resistance of cDNA to S1 nuclease digestion, an enzyme that acts on single stranded DNA oligonucleotides but not on double stranded cDNA (Supplementary Figure S2). The library was then sequenced with a benchtop Oxford Nanopore sequencer (MinION). After base calling, the output was analysed by CircAidMe to identify the inserts and generate the consensus sequence (see Materials and Methods and Figure 1B).

**Figure 2.**
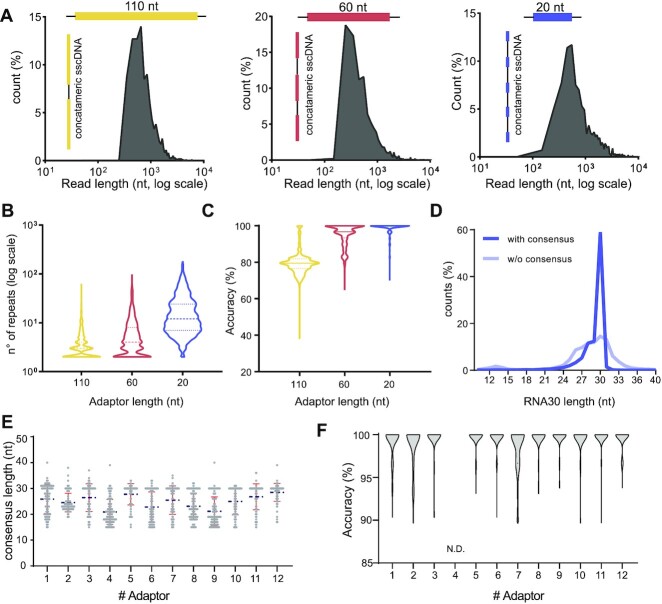
CircAID-p-seq workflow optimization. (**A**) Three different adaptors (ADR110, yellow; ADR60, red and ADR20, blue) were used to perform CircAID-p-seq on a 30 nt long synthetic fragment phosphorylated at the 3′-end. The raw reads length distribution (nucleotides, log scale) is reported as a function of the relative abundance (%) over total reads. (**B**) Violin plot showing the effect of the adaptor lengths on the number of fragment repetitions obtained after RT-RCA (dashed line, median). (**C**) Accuracy of the consensus sequence for the three adaptors (solid line, median). (**D**) Analysis of the 30RNA 3′-P fragments length distribution before (transparent blue) and after (solid blue) consensus generation, using ADR20 as adaptor. (**E**) Length distribution of the consensus sequences obtained from 12 different adaptors used to sequence a 30 nt long synthetic insert. (Blue broken line: mean. Red lines: standard deviation above and below mean). (**F**) Violin plot showing the consensus accuracy of a 30 nt long synthetic fragment obtained with each of the 12 adaptors tested. N.D., not detected, because it did not pass the quality filters for accuracy measurement.

To ensure the robustness of the consensus sequence, a large number of inserts in the concatemer reads is desirable. Thus, we assessed the effect of the ADR length and sequence on the number of generated repeats. We used three adaptors, 20 nt (ADR20), 60 nt (ADR60) or 110 nt (ADR110) long to generate libraries with the same 30 nts synthetic RNA fragment (Figure 2A) (adaptor sequences are listed in Additional file 1). The read length distributions showed a major peak at about 550 nt in all samples, matching the processivity of the RT enzyme previously tested and suggesting that ADR20 generates more tandem repeats than ADR60 or ADR110 (Figure 2A and B). Then, we investigated the impact of the number of repeats on the accuracy (i.e. the percentage of the read not altered by mismatches or indels (39), see Materials and Methods) of the consensus sequence. As expected, the high number of repeats obtained with ADR20 results in a more accurate consensus (Figure 2C) and in a narrower distribution of fragment lengths (Figure 2D). In line with all these observations, to maximize the accuracy of the consensus, we used short ADRs for all further experiments.

Next, we focused on optimizing the sequence composition of ADRs. We designed twelve 24 nts long ADR oligos (Additional file 1), which are predicted to have minimal secondary structure. We pooled them at equimolar concentration for capturing and sequencing the 30 nts synthetic fragment (RNA30-3′P). After sequencing, we evaluated the length and the quality of the consensus sequence of the insert, as well as the relative abundance of each ADR. Almost all adaptors showed a correct consensus length (Figure 2E) and a high accuracy (> 95%) of the insert (Figure 2F) with median of five or more repeats per read (Supplementary Figure S3A). Reads obtained with ADR5 and ADR12 adaptors were more represented than others (Supplementary Figure S3B), suggesting that some of them displayed a higher probability to form RNA:ADR products. Since ADR12 combined a good accuracy with a relatively high read abundance, we used this adaptor in all further experiments.

To investigate whether circAID-p-seq can capture quantitative variations in RNA abundance, we sequenced a mixture of three synthetic RNA fragments (RNA-A, RNA-G and RNA-M) at different molar concentrations. For quantitative analysis, we took into consideration only reverse and ‘hairpin’ reads (Supplementary Figure S4A and S4B and Materials and Methods). The latter are useful to increase the number of repeats (i.e. the accuracy of the consensus) and are generated by a well-known mechanism (36), i.e. the exposed 3' end single-stranded cDNA folds transiently back upon itself to provide a priming point for the polymerase during the second strand synthesis. Our results showed that there is a good agreement (*R*^2^ > 0.98) between the amount of input and the number of consensus sequences obtained for each insert (Supplementary Figure S4C and D). Overall, our results provide evidence that circAID-p-seq (i) can selectively incorporate a mixture of short synthetic RNA molecules bearing a 3′P signature, (ii) is accurate and (iii) is efficiently applicable to the ONT sequencing platform.

### Ribosome profiling with circAID-p-seq

To further confirm the selectivity and efficiency of our method for library preparation in complex biological samples, we chose the framework of Ribo-seq, the sequencing of short RNA fragments protected by ribosomes from nuclease digestion. Ribo-seq provides positional information of ribosomes on transcripts as well as an indication of the RNAs engaged in translation. This experimental setup is intrinsically suitable for our purpose because the endonucleases used, e.g. RNase I, produce 25–35 nt long ribosome protected fragments (RPF), with 3′P ends. Therefore, with circAID-p-seq we can selectively and directly capture only fragments digested by the nuclease, without the need for additional de-phosphorylation steps and without the risk of sequencing RNAs fragments not generated by the enzyme.

**Figure 3.**
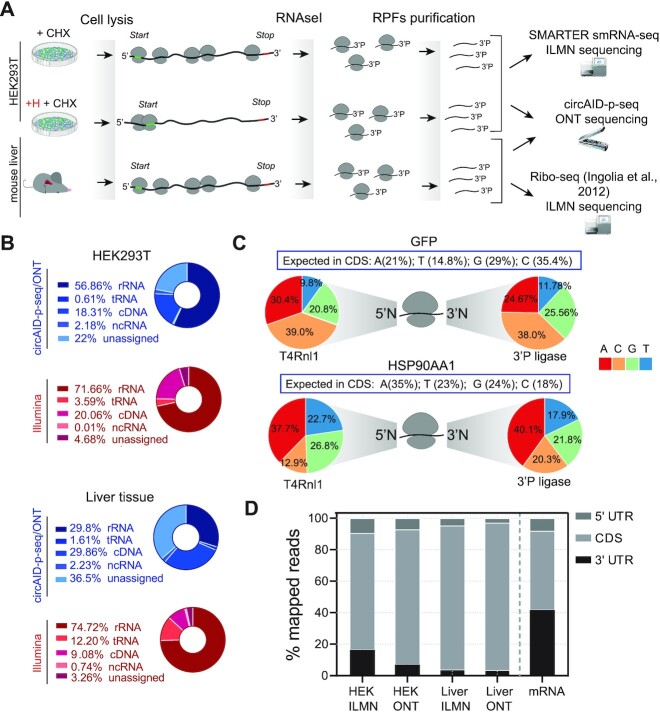
Ribosome Profiling analysis comparison: circAID-p-seq (ONT) vs Ribo-seq (Illumina). (**A**) Schematic representation of ribosome profiling experiments using HEK293T cells and mouse liver tissues. (**B**) Pie chart representing the percentage of reads mapping on coding and non-coding RNAs in HEK293T (top) and mouse liver. (**C**) Frequency of first 5′ nucleotide (5′N) and the last 3′ nucleotide (3′N) calculated for all RPFs detected with circAID-p-seq/ONT within the GFP (top) and the HSP90AA1 (bottom) coding sequence. The pie chart represents the overall percentage of each nucleotide detected at 5′ and 3′ end position of RPFs. 3′P ligase is used for 3′ ligation; T4Rnl1 is used for the intramolecular circularization between the 5′ of RPFs and 3′ of the adaptor. Blue box: percentage of each nucleotide in the entire coding sequence of the transcripts (**D**) Left, percentage of P-sites mapping to the 5′ UTR, coding sequence (CDS), 5′ UTR and 3′ UTR of mRNAs from ONT/circAID-p-seq and Illumina/Ribo-seq data. On the right, theoretical length percentage of each mRNA region (mRNA).

Currently there are no available technologies for selecting and sequencing 3′P RNA fragments, therefore we compared our library preparation method with two established protocols for RPF sequencing: (i) the ligation-free sequencing protocol based on a strand switching approach (29) and (ii) the classical protocol for ribosome profiling (51). Both methods are based on the removal of the 3′P prior to library preparation and sequencing on Illumina (ILMN) platform. We used HEK293T cells to compare circAID-p-seq to a commercial switching approach (SMARTer smRNA-seq, Takara) and mouse liver samples for the standard established protocol of ribosome profiling (Figure 3A).

To determine whether circAID-p-seq can uncover changes in the localization of ribosomes along transcripts, we compared the ribosome footprint distribution in HEK293T cells untreated (H-) and treated (H+) with Harringtonine, a drug known to stall ribosomes at the start codon (52). We obtained 2–5 million raw concatemeric reads per condition by using the CircAID-p-seq libraries, and 47–100 million raw reads using Illumina sequencing. In accordance with Illumina data and with the expected length of RNA fragments covered by ribosomes (53), the length distribution of the consensus sequences, generated by CircAidMe and representing putative RPFs, peaked at about 33 nts in both HEK293T and liver (Supplementary Figure S5). Even if the two platforms have different sequencing depths, we wanted to better understand the concordances between circAID-p-seq and ILMN-based sequencing and library preparation in term of RPFs coverage, number and type of genes identified. We observed a good correlation in RPFs coverage between circAID-p-seq and the two ILMN sequencing methodologies (Spearman's *R* = 0.87 in HEK293T and *R* = 0.91 in mouse liver samples) (Supplementary Figure S6A and B). A current limitation in small RNA sequencing experiments is the over-representation of sequences generated by PCR amplification. According to previous data (54), we observed a discrepancy between ONT and ILMN in highly expressed genes (Supplementary Figure S6C), confirming that PCR duplicates reflects a higher tendency of these genes to produce identical fragments. As circAID-p-seq is a PCR-free protocol, PCR duplicates are not an issue. When we correlated circAID-p-seq data we obtained strong correlations within HEK293T (H + and H-) and within replicates (Pearson's *R* > 0.90) in mouse liver samples (Supplementary Figure S7). As previously observed (55–57), the variability in the number of raw reads within replicates (i.e. genes detected) is likely due to different (i) amount of starting material, (ii) sequencing time and (iii) number of pores available in ONT flow cells (Additional file 4). To confirm the robustness of circAID on single transcripts coverage across replicates, we measured the RPF coverage distribution along the coding sequence (CDS) of four transcript (HbA2, Alb, C4B-201, Hmgcs2). The rationale behind the choice of these transcripts is that the first two (HbA1 and Alb) have, overall, a higher coverage (13 800 TPMs for HbA1 and 4600 TPMs for Alb), compared with Hmgcs2 and C4B (660 TPMs for Hmgcs2 and 630 TPMs for C4B). Our results confirm that the distribution of RPFs within transcripts is consistent among replicates (Supplementary Figure S8).

To finally confirm that circAID-p-seq can detect ribosome footprints, we investigated the number and percentage of reads mapping to protein coding sequences (cDNA). In circAID-p-seq data, 18% of reads from HEK293T and 29% of reads in mouse liver mapped to coding genes. In Illumina data, 20% and 9% of the reads from HEK293T and mouse liver respectively, aligned to coding genes (Figure 3B). We identified a total of 9,419 genes in HEK293T/H- (5,002 with > 10 Transcripts Per kilobase Million, TPM) for circAID-p-seq and 16,754 genes (8820 with > 10 TPM) (Additional file 2) for ILMN. About 55% of genes identified with ILMN were detected also by the ONT sequencing. More than 96% of these genes are in common with ILMN (Supplementary Figure S9A). In line with this result, in circAID-p-seq mouse liver we identified 62% (4838 transcripts with > 10 TPM) of ILMN genes. In this case more than 91% are in common with ILMN (Supplementary Figure S9B). We determined that ILMN covers 0.27% of coding genes per million reads generated, while circAID-p-seq/ONT covers 2.45% of the coding genes per million reads generated. In other words, more than 4000 reads are required to detect a gene in ILMN, while only 130 reads are sufficient with circAID-p-seq. Considering only genes with > 10 TPM, ILMN needs more than 8000 reads/gene while circAID-p-seq requires only 185 reads/gene. Comparable performances were obtained in mouse liver. As a result, even if with ONT the sequencing depth is lower, most of the genes detected by circAID-p-seq (>96%) match the ILMN genes with good read coverage (>10 TPM), reducing the need for deep sequencing. In agreement with this, if we consider all detected genes (>1 count), we observed that the majority of circAID-p-seq data have a gene coverage higher than 1 TPM (with a median of 10 TPM) in both HEK293T cells and mouse liver. Interestingly, genes identified only by ILMN are less covered (median of 0.5 TPM), meaning that they have a low density of RPFs (Supplementary Figure S9C and D). Moreover, very low abundant transcripts are detected less efficiently in circAID-p-seq than with ILMN (Supplementary Figure S9C-D). Overall, these results demonstrate that circAID-p-seq is between four and ten times more efficient in term of number of genes per million of reads with respect to the ILMN sequencing on abundant transcripts.

To further characterize genes identified by the two methodologies, we performed a Gene Ontology (GO) analysis in both HEK293T and mouse liver datasets. We observed similar enriched terms between ILMN and circAID-p-seq/ONT (Supplementary Figure S9E and F), confirming that the most representative transcripts in ILMN were also captured by and were semantically coherent with, circAID-p-seq data.

Of note, CircAID-p-seq showed less reads mapping on tRNA and rRNA than ILMN in mouse liver samples, although a high percentage of unassigned reads is reported (Figure 3B). The lower rRNA contamination suggests that not all tRNAs and rRNAs contaminants derive from cleavage by RNAse I nuclease (i.e. they do not have a 3′P).

To account for ligation biases in the library construction, we analysed the nucleotide composition of the 5′ and 3′ termini of RPFs from the exogenously transfected GFP and the endogenous HSP90AA1, because these transcripts were the two with highest RPF coverage. The nucleotide composition of the first 5′ nucleotide and the last 3′ nucleotide of each RPF was compared to that of the entire CDS. For both ends, we did not observe strong deviations from the CDS (Figure 3C). This result suggests that there are no detectable sequencing biases related to the circAID-p-seq ligation steps (3'Ligase and T4Rnl1).

**Figure 4.**
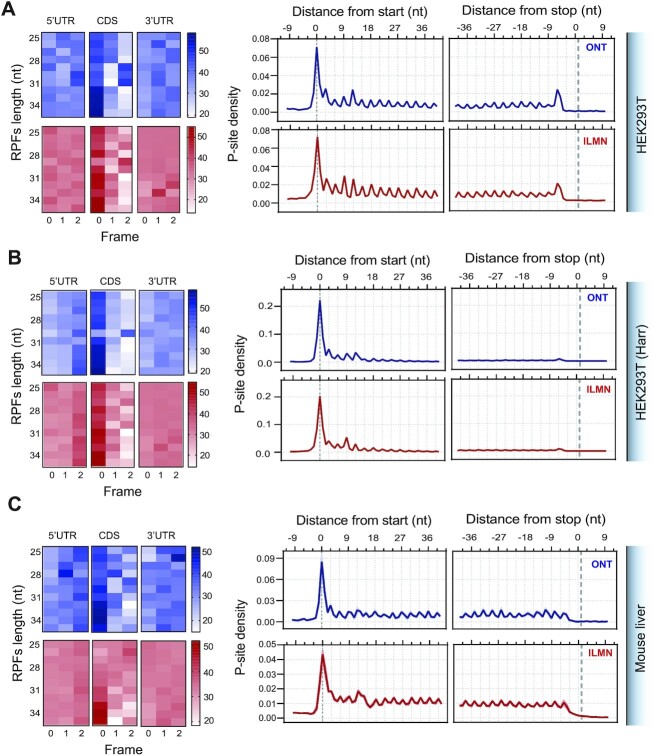
Ribosome footprint data analysis. Percentage of P-sites corresponding to the three possible reading frames (left) along the 5′ UTR, CDS, and 3′ UTR, stratified for read length and metaprofiles (right) showing the density of P-sites around translation initiation site and translation termination site for Riboseq (red) and circAID-p-seq (blue), in HEK293T not treated with Harringtonine (**A**), HEK293T treated with Harringtonine (**B**) and liver tissues (**C**). For liver tissues in C data are mean ± s.e.m. (shadowed line) of *n* = 3 biologically independent samples.

RPFs are expected to be over-represented within the CDS of the mRNAs, where the P-site positions are expected to exhibit trinucleotide periodicity, in contrast to those positioned in 5′UTR and 3′UTR regions. In line with this, a high percentage of the generated consensus sequences mapped to the coding sequence (CDS) within mRNAs (85.7% in HEK293T cells; and 93.4% in mouse liver) (Figure 3D). The percentage of reads mapping in the 5′UTR and 3′UTR was negligible. Finally, the percentage of P-sites in the three possible translation reading frames obtained with circAID-p-seq were in agreement with ILMN data (Figure 4A–C). A comparison between the P-site metaprofiles showed a clear trinucleotide periodicity in all sequencing approaches and samples (Figure 4A–C). Treatment with Harringtonine in HEK293T cells showed a relative increase in the signal at the start codon and a decrease along the CDS (Figure 4B) in both circAID-p-seq and ILMN, confirming the robustness of circAID-p-seq in detecting positional changes of ribosomes. Metaprofiles in mouse liver obtained using only transcripts detected in both library preparation approaches (*n* = 4115; >10 TPMs, Supplementary Figure S10) did not show significant differences between circAID-p-seq and the standard method.

Taken together, these results confirm that circAID-p-seq allows to select and sequence actual ribosome footprints with 3′P ends. Remarkably, circAID-p-seq requires at least 10 times less raw reads than classical ribosome profiling protocols to have a global view on relatively abundant transcripts (TPM > 1). More specifically, circAID-p-seq, coupled with CircAidMe, generates ribosome profiling data consistent with existing methods. Strikingly, cirAID-p-seq has the unique advantages of a higher efficiency and no PCR amplification steps, confirming that it (i) is suitable for ribosome profiling experiments and (ii) is effective for phospho-RNA-sequencing.

## DISCUSSION

To overcome library preparation and sequencing biases due to 3′ modifications of RNA fragments (22,58), we introduced circAID-p-seq, a new approach for library preparation and nanopore sequencing of 2′,3′-cyclic phosphate- and 3′ phospho- terminated RNA fragments.

As a proof of principle, we applied circAID-p-seq to ribosome profiling experiments, which are based on the generation of RNA fragments with a 3′-phosphate generated by endonuclease digestion. We benchmarked our method against commonly used Ribo-seq library preparation protocols that require a dephosphorylation of the 3′ end. In fact, existing approaches are based on PCR steps that can introduce amplification-related issues, although recent methods that include unique molecular identifiers (UMIs) can mitigate this problem (59,60). With circAID-p-seq, no PCR step is required and after RPF purification no gel extraction steps are needed.

CircAID-p-seq is based on two ligation reactions performed by a 3′P ligase and the T4Rnl1. We have strong evidence that in our ribosome profiling data our method is free of any ligation bias because we observed a good balance of the four nucleotides at the 5′-3′ end of the RPFs sequenced, and no positional discrepancy with ILMN methods were detected. Although we did not find detailed reports about 3′P ligase bias, our results on T4Rnl1 are in agreement with previous works, where long incubation times (≥2 h) ensures negligible ligation biases (28,61). We cannot exclude that a combination of sequence length and structure within the complexity of a biological sample will bias, at some extend, the circularization step. Nonetheless the T4 Rnl1 assisted RNA ligation has been demonstrated to be robust and extensively used to produce circular RNA (62). Moreover, successful and efficient intramolecular RNA circularization has been achieved with short (15 nt) RNA strands (63,64) as well as with large viral RNA molecules (>300 nt) (65). Since we focused our attention on a relatively homogenous population of size selected and PAGE purified RNA fragments between 25 and 35 nt, any possible bias related to fragments length were not deeply investigated.

Based on ribosome profiling data, we showed that, circAID-p-seq coupled to ONT sequencing produces high-quality information in terms of (i) number of genes per million of reads and (ii) positional data of the ribosomes along transcripts. In addition, circAID-p-seq/ONT sequencing requires less absolute number of raw reads than classical ribosome profiling coupled with Illumina sequencing to achieve comparable coverage of the translatome. Furthermore, circAID-p-seq showed lower rRNA contamination than other methods, at least in our experiments. These features make the circAID-p-seq library generation extremely useful for genome-wide 3′ phospho-RNA analysis.

If required, an increase in the sequencing depth can be achieved with other ONT sequencing platforms, such as PromethION or GridION. In terms of experimental time, circAID-p-seq combined with CircAidMe allows fast ribosome profiling experiments from sequencing to data analysis (24–48 h). Of note, circAID-p-seq sequencing was performed with the portable and low throughput MinION (ONT) sequencer, affording lower instrumental costs compared to ILMN sequencing. Another advantage of circAID-p-seq/ONT sequencing is that, in the case of samples displaying sequencing problems, the run can be stopped and the flow cell re-used. The main constraints identified for circAID-p-seq are the low sensitivity with low abundance transcripts and the detection of translation events only marginally represented in Ribo-seq data, such as the translation of upstream open reading frames (uORFs), new and rare translation events or ribosome readthrough events (66,67). Future developments of circAID-p-seq need to address the option of workflow multiplexing and of reducing the required amount of input material, which is currently established in more than 3 picomoles of phosphorylated RNA fragments. The relatively high number of unassigned reads is due to the high ONT base calling error (68), that cannot be fully removed by the circAID consensus strategy. Since ONT sequencing is constantly improving with new versions of base calling software and pores, the percentage of circAID-p-seq unassigned reads will be probably lower in the future.

In addition to ribosome profiling, many other RNA footprinting techniques may take advantage of this method. For example, protocols employing endoribonucleases and generating 3′P termini with the aim of characterizing RNA-protein interactions, large RNA-protein complexes (69), and/or the interaction of small molecules with RNA (70).

More importantly, 3′P phosphorylated RNAs are hallmarks of biological processes and can be generated in living cells by toxins (5), ribozymes (4), endonucleases (2,19), the tRNA splicing endonuclease (71), the Ire1 (72), the RNase T2 (21), the RNase L (73) and some CRISPR-associated (Cas) proteins (74). Endogenous 3′P/2′-3′cP terminated RNA fragments are involved in diverse biological processes, such as RNA metabolism (7), rRNA and tRNA biogenesis (8), mRNA splicing (9), unfolding protein response (10) and stress granules production (11). Phosphorylated RNA fragments are also dysregulated in disease conditions, such as cancer (75), viral infection (76) and Amyotrophic Lateral Sclerosis (77) pointing to 3′P/2′-3′cP RNAs as likely and largely unexplored signatures of disease (78).

In conclusion, the combination of circAID-p-seq with ONT allows single molecule, fast and easy detection of biologically relevant 3′-P/cP RNA species with a portable ONT device. CircAID-p-seq is the first phospho-RNA-sequencing library preparation method successfully tested in ribosome profiling experiments and could be used in the near future to better uncover the biological role of 3′-phospho RNA molecules, a still hidden transcriptomic layer in many genome-wide profiles.

## DATA AVAILABILITY

Source code of CircAidMe along with a pre-compiled package and a description of the software are available on GitHub: https://github.com/ms-gx/CircAidMe. The sequencing data have been deposited in NCBI Geo database (accession GSE174754).

## ETHICS APPROVAL AND CONSENT TO PARTICIPATE

All animal tissue was obtained under full licensed approval from the UK Home Office following internal (institutional) and external ethical review.

## Supplementary Material

gkab1158_Supplemental_Files
