# Supplementary material for: Phospho-RNA sequencing with circAID-p-seq
Source: Nucleic Acids Res. 2021 Dec 1;50(4):e23. doi: 10.1093/nar/gkab1158 (PMC8887461; doi:10.1093/nar/gkab1158)
Supplement: gkab1158_Supplemental_Files [file gkab1158_supplemental_files.zip › Supplementary Data and Figures_Del Piano et al 2021_revised 27_10_2021v.docx]

**Supplementary Data includes the following items:**

**Supplementary Figures**

- Supplementary Figure S1: circAID-p-seq workflow validation
- Supplementary Figure S2: circAID-p-seq, second strand synthesis validation
- Supplementary Figure S3: circAID-p-seq adaptor optimization
- Supplementary Figure S4: Quantitative analysis
- Supplementary Figure S5: Ribosome footprints
- Supplementary Figure S6: Data correlation of Illumina and circAID-p-seq/ONT
- Supplementary Figure S7: Data correlation of circAID-p-seq/ONT
- Supplementary Figure S8: Single transcript profiles from mouse liver tissue
- Supplementary Figure S9: Ribosome footprint data analysis
- Supplementary Figure S10: Metaprofiles

**Supplementary Files:**

- Additional file 1: excel file - List of synthetic oligo used for experiments
- Additional file 2: excel file - List of genes and counts for Ribo-seq ILMN and ONT in HEK293T
- Additional file 3: excel file - List of genes and counts for Ribo-seq ILMN and ONT in mouse liver tissues
- Additional File 4: excel file - Liver samples: starting RNA amount and run details

**
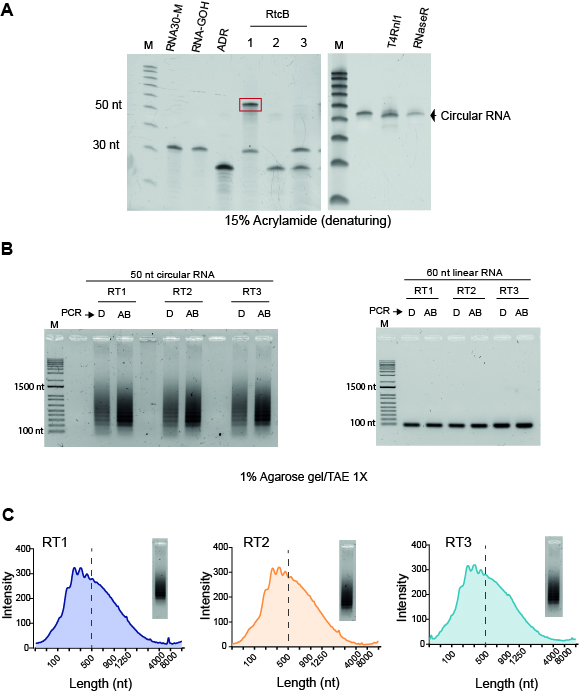
**

Figure S1. **circAID-p-seq workflow validation. (A)** TBE-urea PAGE gels showing the 3′P-mediated ligation of a synthetic 30 nt long RNA-3’P and a 24 nt long adaptor (ADR12, left panel). Lane labels indicate: RNA30-M, synthetic RNA oligo of 30 nt with a 3’P; RNA-GOH synthetic RNA oligo of 30 nt with a 3’OH; ADR, adaptor; 1, 3′P- ligation reaction between ADR12 and RNA30-M (red box shows the reaction product); 2, 3′P- ligation reaction only with ADR12 without any RNA fragment (negative control); 3, 3′P- ligation reaction between ADR12 and RNA30-GOH. On the right panel, RNA-3’P-ADR product extracted and loaded on the TBE-urea gel again, followed by circularization with T4 Rnl1 and digestion with RNase R. Note that only 1/10 of the total RNA products were loaded for each step to not overload the lane. **(B)** Agarose gels showing PCR products obtained from circular (left panel) and linear (right panel) cDNA retrotranscribed using three different RT enzyme (RT1,RT2,RT3), by two different Taq enzymes: D, dreamTaq (Thermo Fisher); AB, Super AB Taq (AB Analitica). **(C)** Representative densitometry traces of the agarose gel, as in (B), show the distribution of multimeric cDNA resolved by size, using three different RT enzyme. The densitometry analysis was performed in ImageJ.


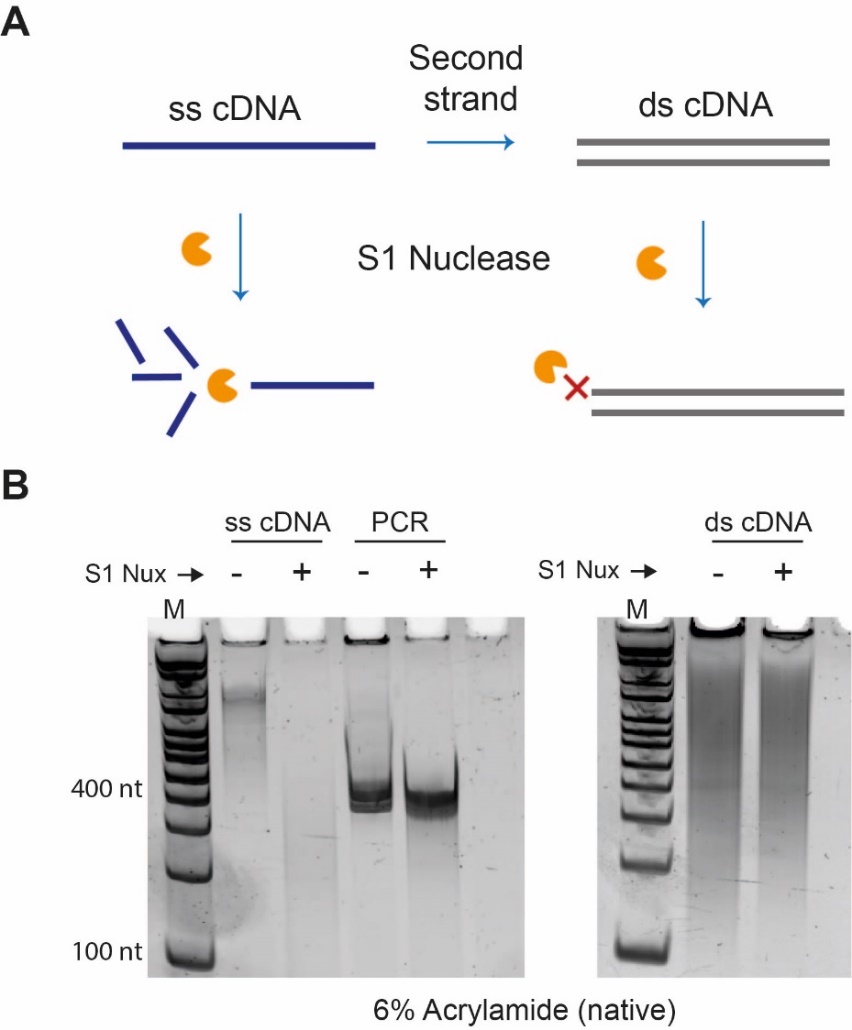


Figure S2. **circAID-p-seq, second strand synthesis validation.** **(A)** schematic representation: S1 nuclease digests single-stranded cDNA but not double-stranded cDNA. **(B)** TBE-urea PAGE gels showing the effect of S1 nuclease (S1 Nux) treatment (+) compared with no treatment (-) on the single-stranded cDNA and a PCR products (left). On the right, the double-stranded cDNA obtained with circAID-p-seq with (+) and without (-) S1 treatment.


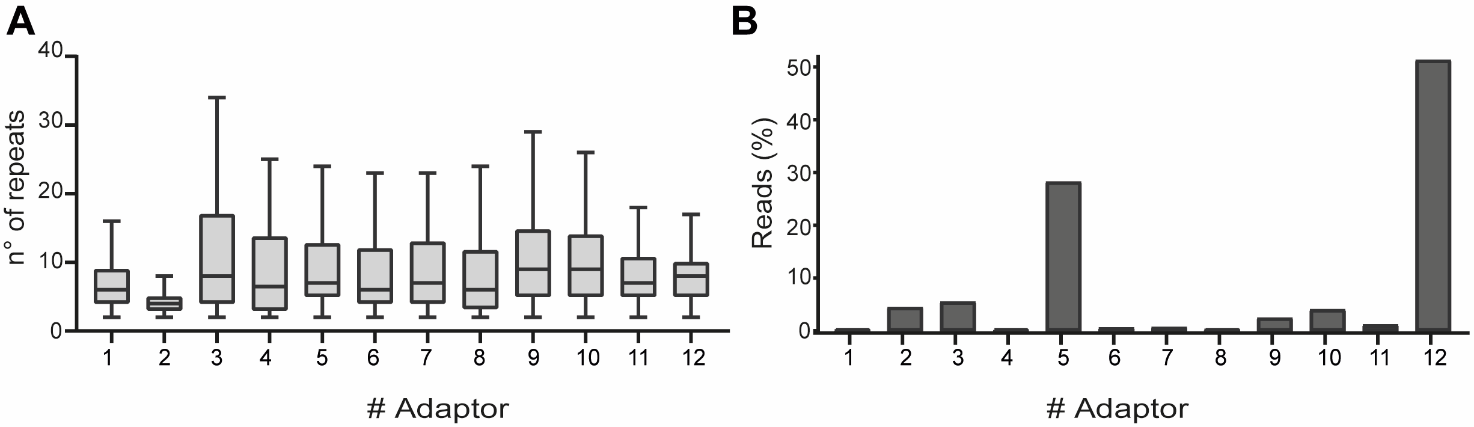


Figure S3. **circAID-p-seq adaptor optimization.** Output from an equimolar pool of 12 different adaptors. **(A)** Number of repeats obtained from each adaptor. **(B)** Percentage of reads obtained from each adaptor.


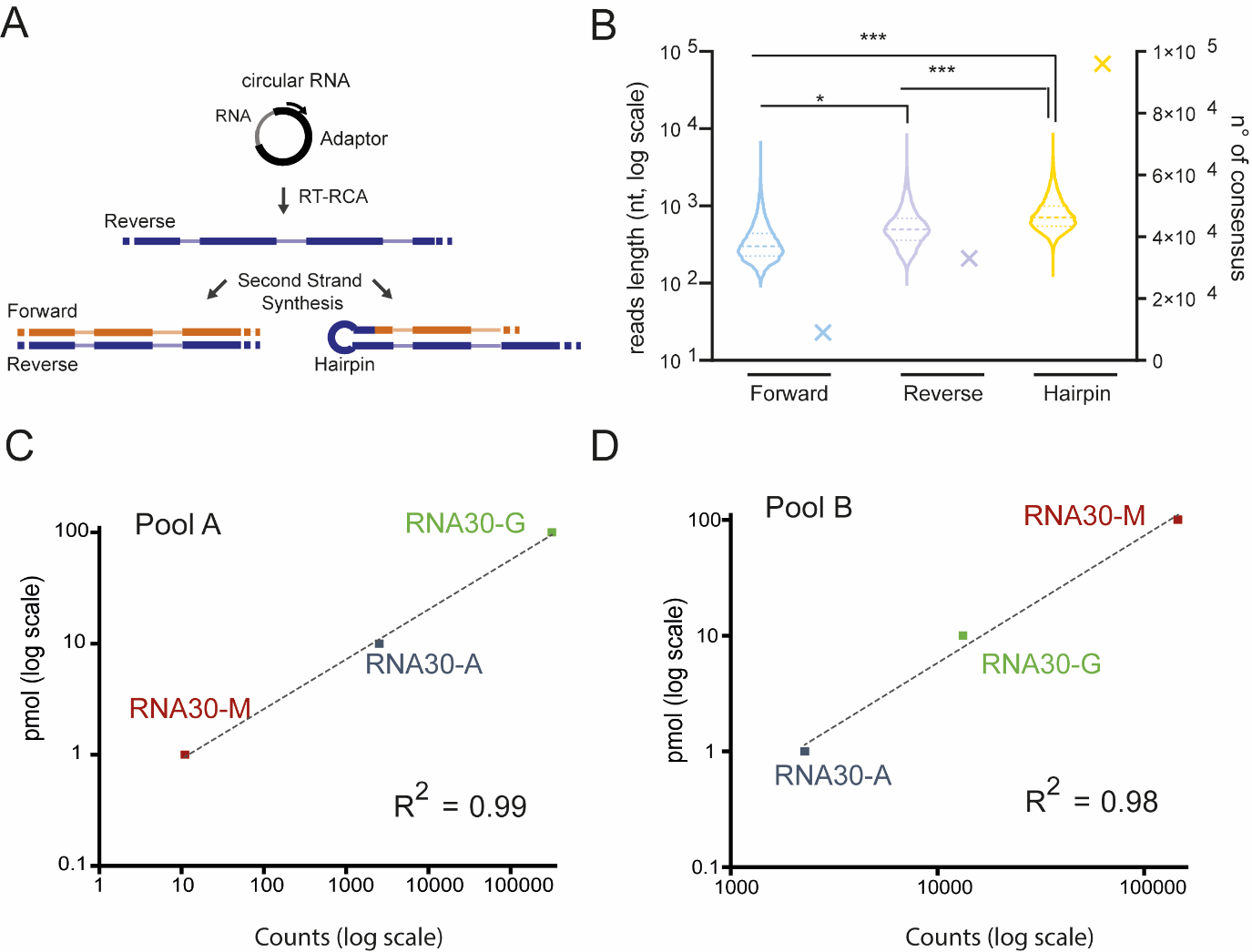


Figure S4. **Quantitative analysis. (A)** Schematic representation of reverse (blue), forward (orange) and hairpin strand, formed during circAID-p-seq steps of RT-RCA and second strand synthesis. **(B)** Violin and dot plot reporting the reads length counts (dashed line: median). The crosses report the number of consensus sequences generated after circAidMe analysis for each strand. Significance was tested with Wilcoxon test P-value (*=P < 0.05; ***= P  ≤ 2.792e-13) **(C)** Correlation between read counts and expected abundances (pmol) for Pool A **(**RNA30-M, RNA30-A and RNA30-G, mixed at 1:10:100 picomolar ratio respectively). **(D)** Correlation between read counts and expected abundances (pmol) for Pool B **(**RNA30-A, RNA30-G and RNA30-M), mixed at 1:10:100 picomolar ratio respectively. The GC content is 40% (RNA30-M, RNA30-A) and 63% (RNA30-G) and no specific RNA motifs were selected. Squared Pearson correlation coefficients (R^2^) are reported.


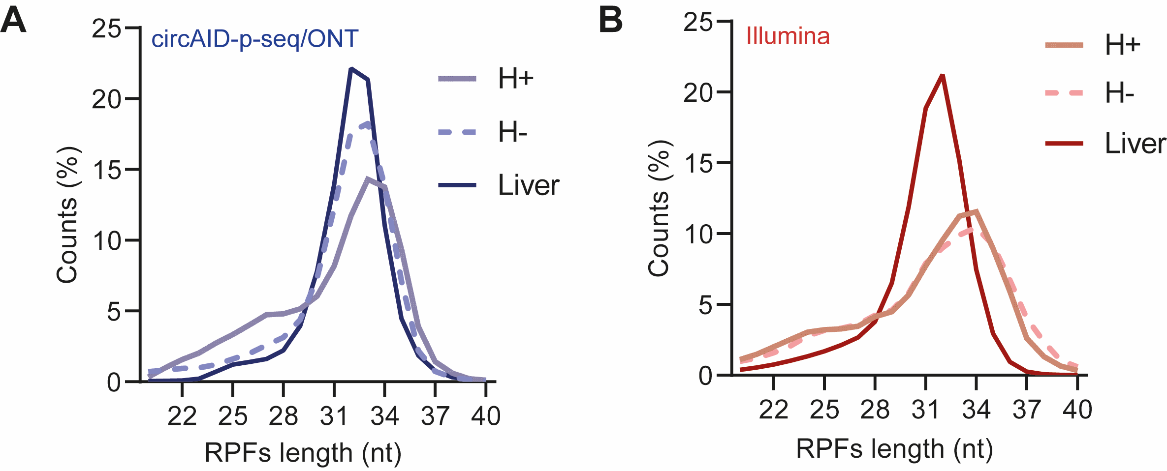


Figure S5. **Ribosome footprints.** Ribosome protected fragments (RPFs) length distribution obtained from cells treated (H+) or not (H-) treated with Harringtonine and from liver tissues using circAID-p-seq library prep **(A)** and the two Illumina Ribo-seq methods **(B).**


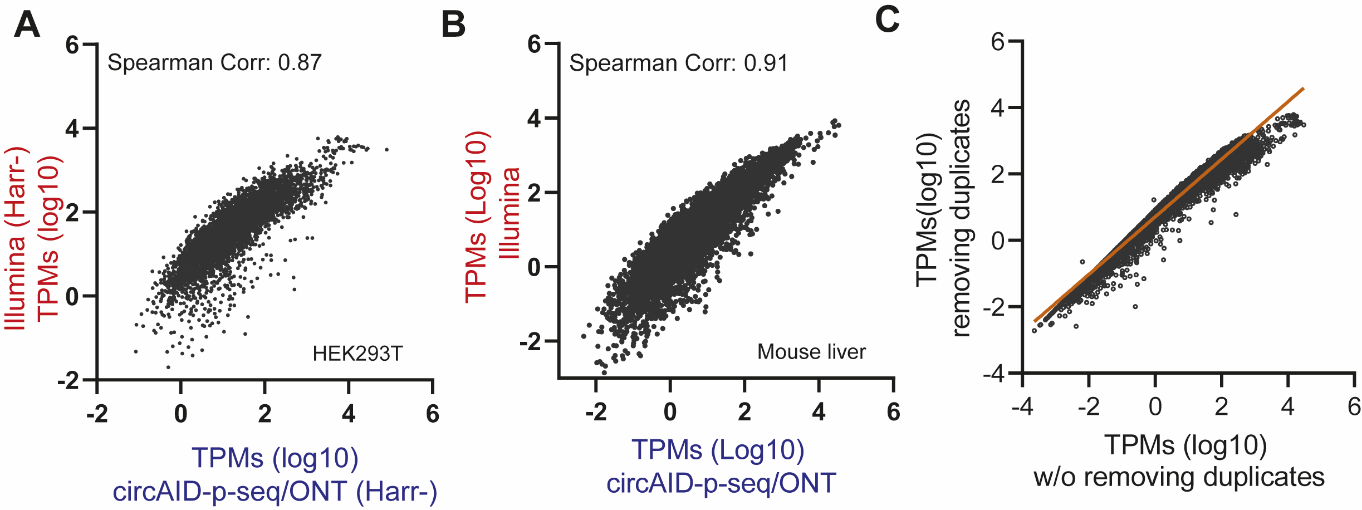


Figure S6 **Data correlation of Illumina and circAID-p-seq/ONT. (A)** RPF coverage correlation between circAID-p-seq/ONT and ILMN, in HEK293T experiment. HEK293T were transfected with GFP in different days for circAID-p-seq/ONT and ILMN sequencing respectively **(B)** RPF coverage correlation between circAID-p-seq/ONT and ILMN, in mouse liver. Data are mean of n= 3 biologically independent samples. For all data, Spearman’s rank correlation are reported in figure. All genes are filtered for more than 1 count. (**C**) RPF coverage correlation between Illumina with or without PCR duplicates removal, in HEK293T experiment. Orange lane: best linear fit (y = 1.15x). These data confirm what is reported in Fu et al., 2018, BMC genomics.


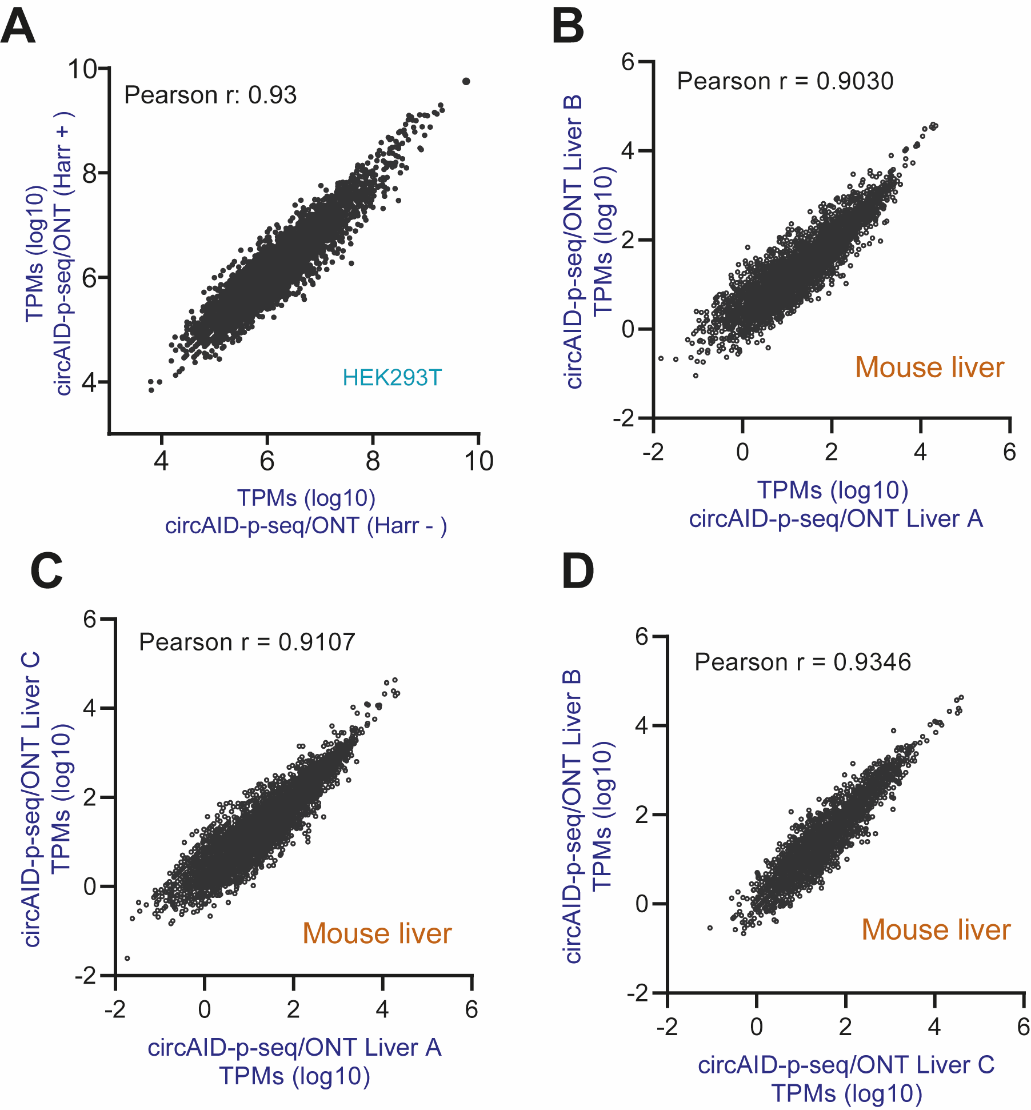


Figure S7 **Data correlation of circAID-p-seq/ONT. (A)** RPF coverage correlation between HEK293T treated and not treated with Harringtoine using circAID-p-seq/ONT method. **(B-D)** RPF coverage correlation between the three biological replicates of mouse liver tissue (Liver A, B, C) using circAID-p-seq/ONT method. For all data, Pearson linear correlation are reported in figure. All genes are filtered for more than 1 count.


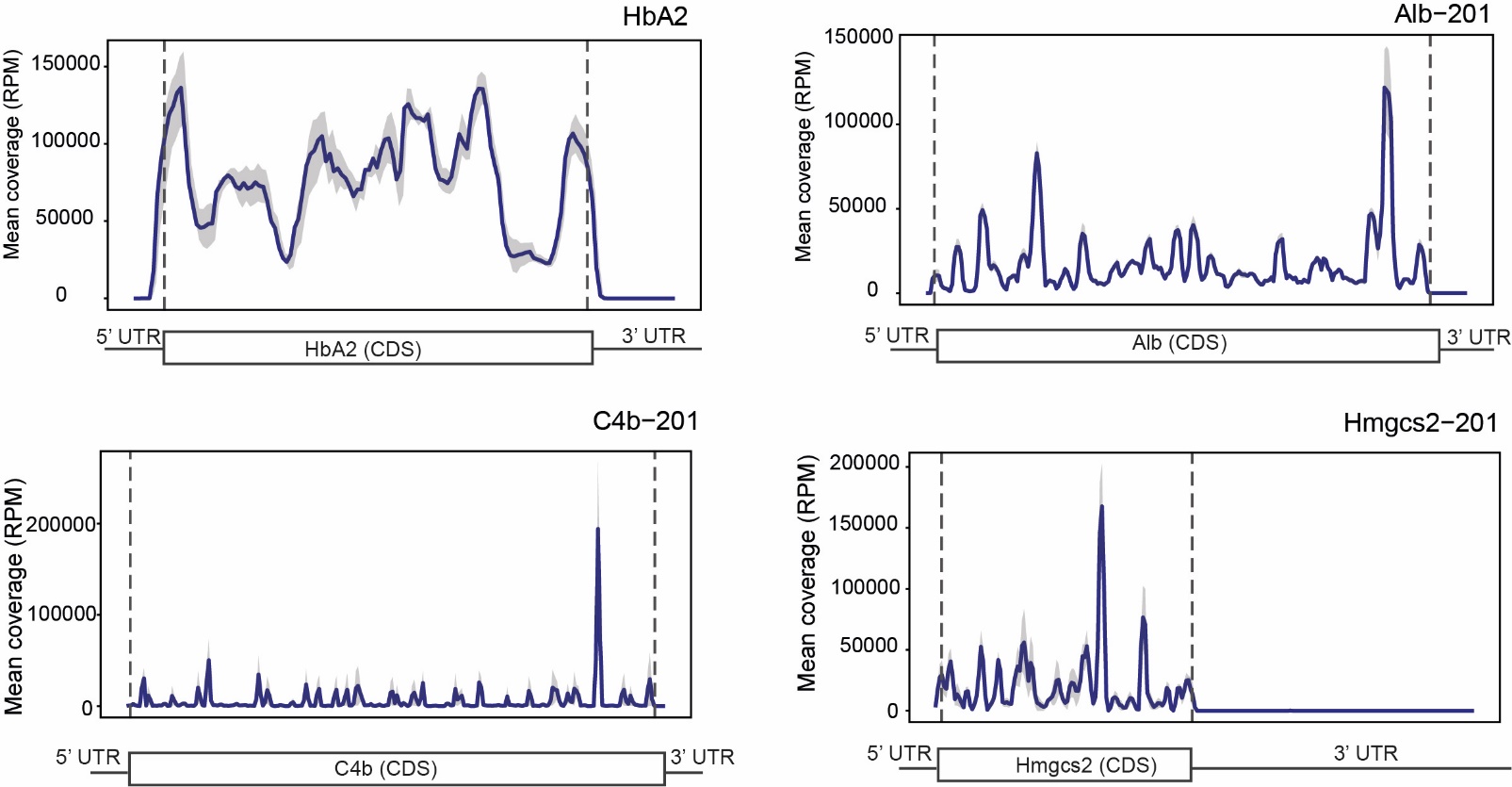


Figure S8 **Single transcript profiles from mouse liver tissue.** Representative RPF coverage tracks for four different transcripts detected in circAID-p-seq/ONT. Results are shown as the mean ± s.e.m. of n = 3 biologically independent samples (grey shadow). On the bottom of each profile the schematic representation of the coding sequence (CDS) and untranslated regions (UTRs) is shown.


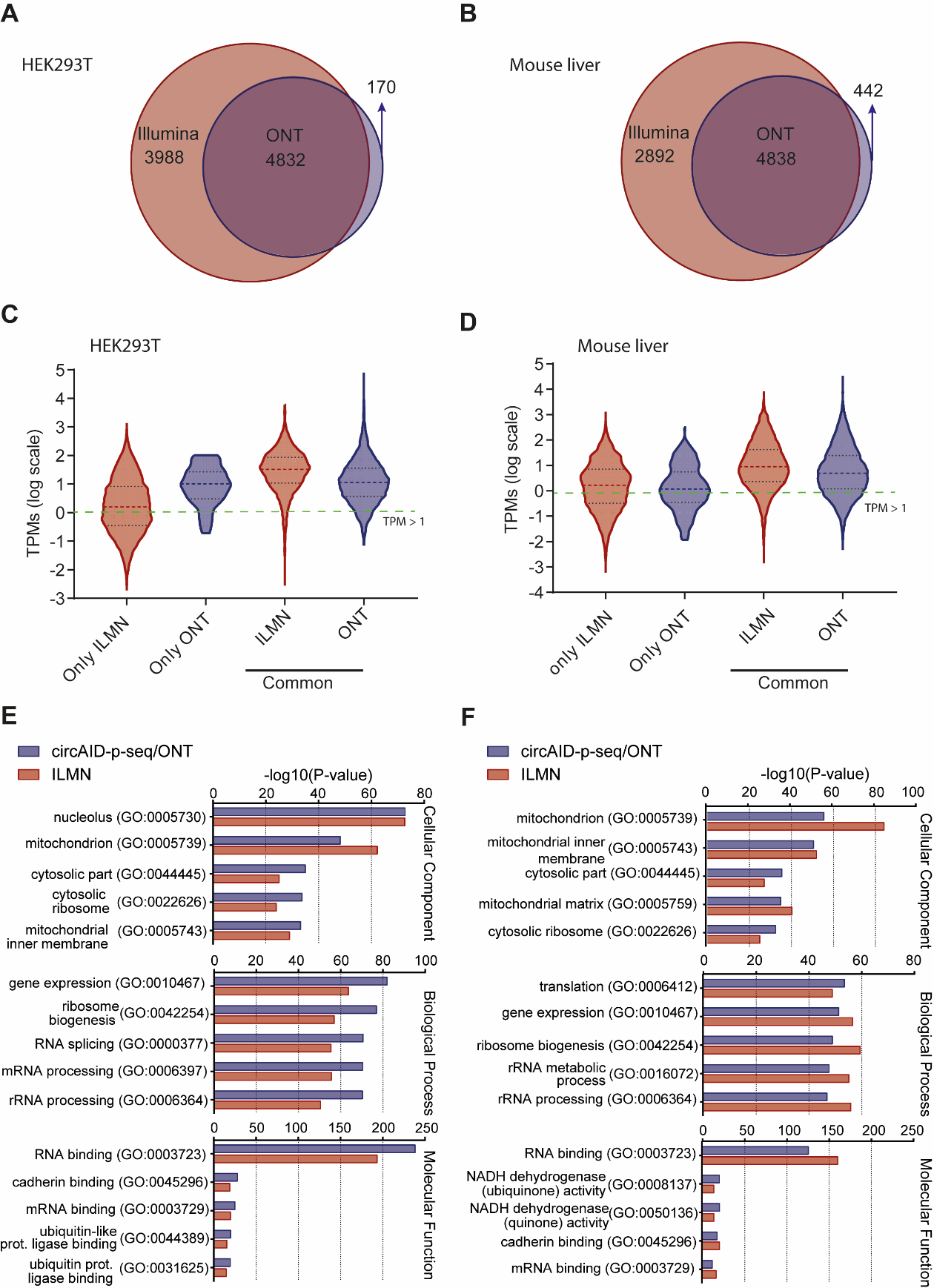


Figure S9. **Ribosome footprint data analysis. (A)-(B)** Venn diagram showing the number of common and unique genes (TPMs >10), between ILMN and circAID-p-seq/ONT, in (A) HEK293T and (B) mouse liver. **(C)-(D)** Violin plot showing the distribution of TPMs of genes identified (>1 count) with Illumina (ILMN) and circAID-p-seq/ONT (ONT), in (c) HEK293T and (**d**) mouse liver. Dashed line=median; dotted line=quartiles. For mouse liver tissues data are mean of n= 3 biologically independent samples. Green broken line, TPM > 1 threshold. **(E)-(F)** GO analysis for genes (TPMs >10) detected by circAID-p-seq/ONT (blue) and ILMN (red) in (E) HEK293 and (F) mouse liver. Top-5 enriched term for each category are sorted by value of –Log10(p-value).


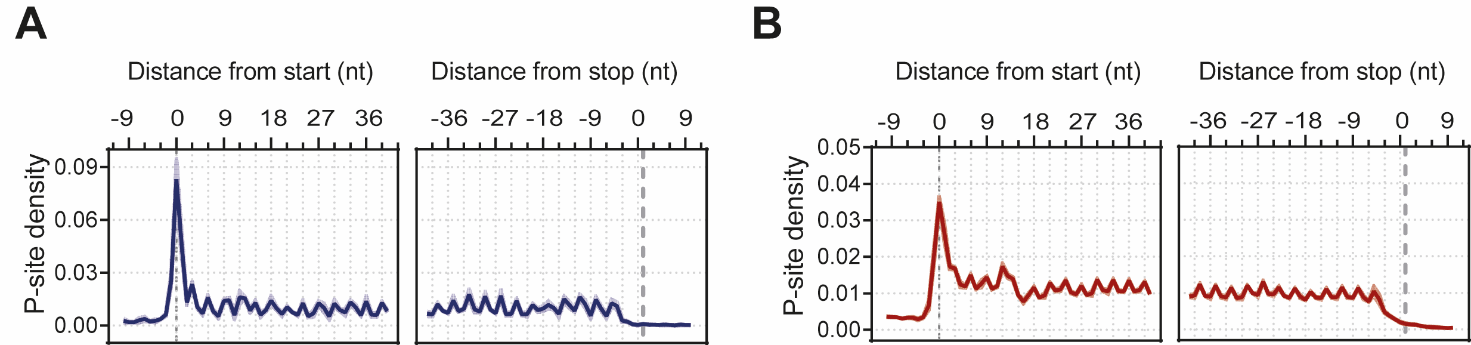


Figure S10. **Metaprofiles**. Metaprofiles for mouse liver tissue data showing the density of P-sites around translation initiation sites and translation termination sites for circAID-p-seq/ONT (**A**) and Riboseq/ILMN (**B**), using only common transcripts detected by both technology (n = 4115; > 10 TPMs). Data are mean ± s.e.m. of n = 3 biologically independent samples (shadow).
